# Rumen methanogens and mitigation of methane emission by anti-methanogenic compounds and substances

**DOI:** 10.1186/s40104-017-0145-9

**Published:** 2017-01-26

**Authors:** Amlan Patra, Tansol Park, Minseok Kim, Zhongtang Yu

**Affiliations:** 1 Department of Animal Sciences, The Ohio State University, 2029 Fyffe Road, Columbus, OH 43210 USA; 2 Department of Animal Nutrition, West Bengal University of Animal and Fishery Sciences, 37 K. B. Sarani, Belgachia, Kolkata, 700037 India; 3 Animal Nutrition and Physiology Team, National Institute of Animal Science, Rural Development Administration, Wanju, 55365 Republic of Korea

**Keywords:** Anti-methanogenic compound, Methanogen, Mitigation, Protozoa, Rumen

## Abstract

Methanogenic archaea reside primarily in the rumen and the lower segments of the intestines of ruminants, where they utilize the reducing equivalents derived from rumen fermentation to reduce carbon dioxide, formic acid, or methylamines to methane (CH_4_). Research on methanogens in the rumen has attracted great interest in the last decade because CH_4_ emission from ruminants contributes to global greenhouse gas emission and represents a loss of feed energy. Some DNA-based phylogenetic studies have depicted a diverse and dynamic community of methanogens in the rumen. In the past decade, researchers have focused on elucidating the underpinning that determines and affects the diversity, composition, structure, and dynamics of methanogen community of the rumen. Concurrently, many researchers have attempted to develop and evaluate interventions to mitigate enteric CH_4_ emission. Although much work has been done using plant secondary metabolites, other approaches such as using nitrate and 3-nitrooxy propanol have also yielded promising results. Most of these antimethanogenic compounds or substances often show inconsistent results among studies and also lead to adverse effects on feed intake and digestion and other aspects of rumen fermentation when fed at doses high enough to achieve effective mitigation. This review provides a brief overview of the rumen methanogens and then an appraisal of most of the antimethanogenic compounds and substances that have been evaluated both in vitro and in vivo. Knowledge gaps and future research needs are also discussed with a focus on methanogens and methane mitigation.

## Background

The unique environment (e.g., relatively rapid passage rate, readily available carbon dioxide (CO_2_) and hydrogen (H_2_)) in the rumen helps assemble a community of archaea distinct to that of other anoxic habitats. Nearly all of these archaea are methanogens, most of which are hydrogenotrophic rather than acetoclastic methanogens even though ruminal acetate reaches high concentrations. Rumen methanogens scavenge H_2_ and CO_2_ produced by other fermentative members of the ruminal microbiome, producing CH_4_. Formic acid and methylamines produced by other rumen microbes are also available as substrates for rumen methanogens [1]. Therefore, methanogens interact with other ruminal microbes, including protozoa [2], bacteria [3], and fungi [4], through interspecies H_2_ transfer. Overall, such interaction benefits the rumen fermentation as it prevents H_2_ accumulation and feedback inhibition. Most of the methanogens live freely in rumen liquid or as members of the biofilm adhering to feed particles, whereas a small portion of the ruminal methanogens are symbionts, either ectosymbionts or endosymbionts [5]. In co-cultures, a hydrogenotrophic methanogen shifts fermentation towards acetate, increasing ATP yield and growth of cellulolytic bacteria [6]. In vivo studies also showed that inhibition of methanogens decreases acetate: propionate ratio, reflecting a shift of fermentation towards more reduced volatile fatty acids (VFA) than towards acetate [7–9]. Rumen CH_4_ emission accounts for about 17% of the global CH_4_ emission [10]. About 2–12% of the ingested feed energy is also lost as CH_4_ [11]. Therefore, ruminal methanogens have attracted much research interest in the past decade with an aim to understand their diversity and community structure, relationship with other ruminal microbes and with feed efficiency, CH_4_ emission, and responses to dietary interventions that were intended to mitigate ruminal CH_4_ emission. Enabled by comprehensive analysis using next generation sequencing (NGS) technologies, new information in the aforementioned aspects has been learned, but contradicting results are also reported, and critical gaps in our knowledge remain. Here we review the current understanding of ruminal methanogens, with an emphasis on protozoa-associated methanogens (PAM) and the responses of ruminal methanogens to anti-CH_4_ compounds and substances. Future research needs are also discussed.

## Overview of methanogens present in the rumen

**Fig. 1:**
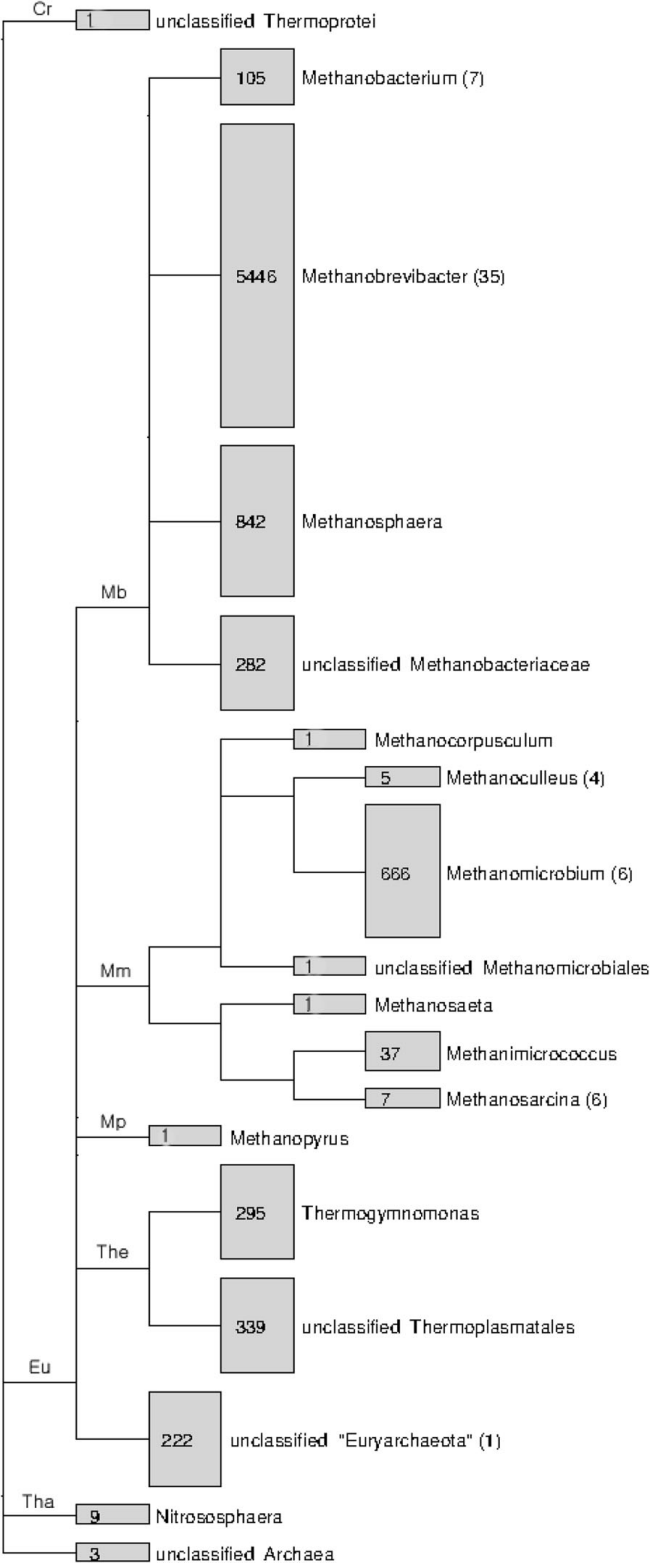
A taxonomic tree showing rumen archaea. A total of 8623 sequences of rumen archaea were retrieved from the RDP Release 11 (Update 3). Information on sequences recovered from isolates was indicated in parentheses. Cr, *Crenarchaeota*; Eu, *Euryarchaeota*; Tha, *Thaumarchaeota*; Mb, *Methanobacteria*; Mm, *Methanomicrobia*; Mp, *Methanopyri*; The, *Thermoplasmata*

The diversity of the rumen methanogens is much smaller, and their diversity is much lower than that of rumen bacteria, with archaeal SSU rRNA only accounting for 6.8% of rumen total SSU rRNA [12]. Archaea in the rumen is represented by <3.3% of the total rRNA (both 16S and 18S) therein. Only eight species of ruminal methanogens have been isolated into pure cultures: *Methanobacterium formicicum*, *Methanobacterium bryantii*, *Methanobrevibacter ruminantium*, *Methanobrevibacter millerae*, *Methanobrevibacter olleyae*, *Methanomicrobium mobile*, *Methanoculleus olentangyi*, and *Methanosarcina barkeri* [13]. Recently, five new species were isolated, including *Methanobrevibacter boviskoreani* (isolated from the rumen of Korean native cattle) [14], *Methanobacterium beijingense* (isolated from the rumen of goat), *Methanoculleus marisnigri* (isolated from the rumen of Indian crossbred cattle), *Methanoculleus bourgensis* (isolated from the rumen of Holstein cattle), and *Methanosarcina mazei* (isolated from the rumen of Korean Hanwoo cattle) (based on the RDP database). One *Thermoplasmatales*-like pyrrolysine-dependent archaeon BRNA1 was isolated from bovine (GenBank access number: CP002916). Collectively, 16S rRNA gene sequences from cultured methanogens only accounted for approximately 0.7% of the total archaeal sequences of rumen origin, and several taxa do not have a single cultured representative (Fig. 1). Most of the isolates are members of the family *Methanobacteriaceae*. Compared to other anaerobic habitats where >100 species of methanogens of 28 genera have been isolated, the diversity and species richness of ruminal methanogens are quite low, reflecting the highly selective ruminal environment for methanogens. In addition, sequenced ruminal 16S rRNA gene clones shared >95% sequence similarity with that of *Methanobrevibacter gottschalkii*, *Methanobrevibacter thaueri*, *Methanobrevibacter smithii* and *Methanosphaera stadtmanae* [15, 16], indicating that these species may be common ruminal methanogens.

Much of the ruminal methanogen diversity was characterized by 16S rRNA gene sequences. The RDP Release 11 (Update 3) contains 8623 archaeal 16S rRNA gene sequences of rumen origin. These sequences were generated using the Sanger sequencing technology, which produces higher sequence accuracy than NGS technologies, in 96 separate studies including 48 unpublished studies. About 90% of these sequences were assigned to methanogens (Fig. 1). These sequences were classified to 10 known genera, with *Methanobrevibacter* being represented by 63.2% of all the sequences followed by *Methanosphaera* (9.8%), *Methanomicrobium* (7.7%), and *Methanobacterium* (1.2%). It should be noted that *Methanocorpusculum* and *Methanosaeta* were each represented by only one sequence. These two genera of methanogens are probably not residents of the rumen. The order *Thermoplasmatales*, which was previously referred to as the rumen cluster C (RCC) group, is represented by 7.4% of the total archaeal sequences. One apparent discrepancy is the good representation of cultured species of *Methanobacterium* and relatively small proportion of sequences classified to this genus. The opposite holds true for the genus *Methanosphaera*. Understanding such discrepancies and isolation and characterization of *Thermoplasmatales*-like methanogens will help further advance the microbial biology of rumen methanogens.

## Free-living ruminal methanogens

Most of the methanogens are not associated with ruminal protozoa or fungi [17], which is reflected by the smaller number of 16S rRNA gene sequences recovered from protozoa than from rumen content or fluid (461 vs. 8162 16S archaeal rRNA gene sequences archived in RDP). It should be noted that this result could also arise from the difficulty associated with obtaining archaeal DNA from protozoal cells. No sequence assigned to *Methanobacterium* has been recovered from rumen protozoa, leading to speculation that species of *Methanobacterium* are probably not PAM. In contrast, a significant portion (32.8%) of the *Methanobrevibacter* sequences archived in RDP was recovered from protozoa. *Methanosphaera* was also thought to be free-living. However, considering that *Methanobrevibacter* accounts for at least 65% of the rumen methanogens, the significant portion of the *Methanobrevibacter* sequences recovered from rumen protozoa may simply reflect the probability of sequence recovery, rather than a selective association between rumen protozoa and *Methanobrevibacter*. It should be noted that the majority of “free-living” methanogens are actually integrated into the biofilm on the surface of feed particles where H_2_-producing bacteria actively produce H_2_ [18]. Being protected by the biofilm, these methanogens may not be inhibited as much as the free-living peers by anti-methanogenic inhibitors.

## Methanogens associated with rumen protozoa

Most species of the rumen ciliate protozoa contain hydrogenosomes, a unique type of membrane-bounded organelles producing H_2_ by malate oxidization [19]. These organelles can attract some methanogens as endosymbionts [13]. Hydrogen generated by rumen protozoa could be utilized by PAM, which benefits both parties [20]. Methanogens have been observed internally [21] and externally [2, 22]. Through feeding or fasting of sheep and by flushing the sheep rumen with N_2_ or H_2_ gas, Stumm et al. [23] showed that the frequency of methanogens associated ectosymbiotically was affected by the relative contribution of H_2_ production by rumen ciliates and H_2_-producing bacteria. This is expected, but it remains to be determined if the species of methanogens associated endosymbiotically with rumen ciliates can also be affected. Based on fluorescence *in situ* hybridization (FISH) analysis, about 16% of the rumen ciliates contained methanogens inside their cells [24]. A possible explanation for the low incidence is that the intracellular association may be transient rather than permanent. However, early studies indicated that rumen ciliates do not have endosymbiotic methanogens though they might have ectosymbiotic methanogens [19, 25, 26]. The difficulty in distinguishing engulfed methanogens from true endosymbiotic methanogens presents a challenge to determining if rumen ciliates possess true endosymbiotic methanogens and bacteria.

Some studies have attempted to identify PAM (Table 1). Because of the labor-intensive procedures involved, PAM are mostly identified using DNA-based methods, and only one strain of methanogen (isolates MB-9; related to *Methanobrevibacter ruminantium*) has been reported to be associated with a ciliate fraction of the rumen of sheep [27]. Among the methanogen sequences of rumen origin archived in the RDP database (Release 11, Update 3), only a very small proportion (5.3%) was recovered from washed protozoa cells. These sequences were derived from a limited number of studies [28–33]. *Methanobrevibacter* and *Methanomicrobium* were the first and the second largest genera reported to be PAM, and they accounted for 32.8% and 23.0% of the total PAM sequences, respectively. *Methanomicrobium* is better represented in the PAM sequences (23.0%) than in the total archaeal sequences (7.7%), so is *Thermoplasmatales* (22.1% vs. 7.4%). Species of both taxa may be among the predominant PAM. It should be cautioned that the above results may be biased because only a small number of PAM sequences were obtained from selected protozoa [13]. Besides, the PAM sequences may be contaminated with sequences of non-PAM. Therefore, future studies are needed to characterize PAM using methods that can eliminate possible contamination with non-PAM.

**Table 1:** Techniques used to define the association between rumen protozoa and methanogens in 14 references

Techniques	Description	Methanogen population	Host ciliate	Animals & Diet & Sampling	Reference
Culture-based enumeration	MPN numbers of methanogens per ciliate cells were measured after each time points after feeding	Maximum number of methanogens are detected 1 h after feeding (10^3^ to 10^4^ MPN/cell)	*Polyplastron* *Ophryoscolex* *Isotricha* *Entodinium* spp.	- Animals : Sheep - Diet : Mixed diet - Sampling : 0, 1, 2 and 3 h after feeding (in vitro culture)	[172]
Culture-based isolation & repeated washing + RFLP	1. Isolation of culturable methanogens from ciliate fraction on selective media 2. Retrieve the 16S rRNA sequences from washed ciliate fraction	isolates MB-9 - > ^a^ *Mbb. ruminantium* *Mbb. smithii* related sequences were dominant	Ciliates fraction	- Wethers - Mixed diet (twice a day) - 1 h after morning feeding	[27, 29]
Defaunation (DGGE + qPCR)	Postinoculation of various protozoal fauna in defaunated sheep and notify the different archaeal phylotypes depends on the specific groups of rumen ciliates	Predominant associated archaea species; Isotrichidae—*Mbb. smithii* *P. multivesiculatum*—*Mbb. bryantii*, *Mbb*. *stadtmanae*, and *Mbb. ruminantium* Holotrichs—uncultured archaea	4 different types of fauna	- Wethers - Corn silage + SBM - Before morning feeding	[32]
Defaunation (DGGE + qPCR)	Microbial population shift after long-term defaunation (methanogenic archaea & fibrolytic bacteria)	Abundance of methanogens ↑, w/no difference on diversity in the absence of protozoa	Entodiniomorphs (97%) Holotrichs (3%)	- Wethers (in vivo) - Mixed diet (once daily) - Just before feeding	[173]
Defaunation (DGGE)	Short & long-term defaunation effect on the association between rumen protozoa and methanogens	Defaunated and faunated samples from the liquid phase were placed in an independent cluster (DGGE)	3.8 × 10^6^/ml ciliate cells (95% Entodiniomorphs, 1.2% Isotricha and 2.9% Dasytricha)	- Wethers - Mixed diet (twice a day) - 3 h after morning feeding	[174]
Defaunation (qPCR + TRFLP)	Protozoal fractions (w/nylon meshes of 80, 60, 45, 35, 20 and 5 μm pore diameters) were made by size fractionation.	No difference of methanogens abundance in- and out-side of ciliate cells. Holotrichs has different methanogen community compared to the total protozoal fraction. T-RFLP—Clear differences between PAM & free-living methanogens. Low similarity among each protozoal fractions	Holotrich protozoa & total protozoa fraction	- Sheep - Mixed diet (twice a day) - Before morning feeding	[17]
Repeated washing	Washed protozoa fraction from monofaunated rumen fluid was used for DNA extraction. Phylogenetic analysis was done with sequences. The associated methanogens are highly correlated with the species in the rumen fluid.	All sequences showed high similarity to the family Methanobacteriaceae	*Isotricha prostoma* *Eudiplodinium maggii* *Polyplastron* *multivesiculatum*	- Sheep’s rumen (monofaunated)	[28]
Repeated washing + qPCR	mcrA & 16S rRNA gene was amplified from washed protozoal fraction. Construction of clone library with amplicons for phylogenetic analysis qPCR for quantification of each methanogenic group (Mbb, ^b^Mm, ^c^RCC)	*Methanomicrobium* spp. was mostly found in free living environment Mbb (free living-18, PAM-34%) Mm (free living-25, PAM-17%) RCC (free living-58, PAM-48%) Forage - > high grain diet (RCC↓, Mbb↑)	Ciliates fraction	- Heifers - Forage fed - > highconcentrate diet/d - 1 h prior to feeding	[175]
Single cell isolation	Extracellular microbes were removed by antibiotics treatment. 16S rRNA gene sequences were amplified from the isolated single cells of each protozoal species and sequenced.	*Methanobrevibacter* sp. was the most abundant genus among three ciliates. Minor detection of *Methanomicrobium* sp. and RCC group were found.	*Polyplastron* *multivesiculatum* *Isotricha intestinalis* *Ophryoscolex purkynjei*.	- Goat’s rumen (in vitro)	[30]
Single cell isolation	Methanogen population distributed to each protozoal species analyzed by single cell isolation followed by sequencing of SSU rRNA genes	Retrieved 20 novel sequences had low identity to the known sequences in the databases. *Methanimicrococcus baltticola & Mm. mobile* were the most related known species among the protozoa species.	*Ophryoscolex caudatus* *Metadinium medium* *Entodinium furca* *Diplodinium dentatum*	- Sheep, Cow and Goat's rumen + Sheep’s rumen (in vitro)	[31]
Single cell isolation + DGGE	16S rRNA gene was amplified from the isolated *Entodinium caudatum* cells and applied to DGGE	Only one DGGE band was shown from isolated single cell. The sequence only found from isolated Ento cell not in the total DNA.	*Entodinium caudatum* (Long-term in vitro cultured)	- Sheep’s rumen (in vitro)	[176]
FISH probing	FISH was applied to detect prokaryotes colonized in various protozoal species	*D. ruminantium* (archaea (−)) *Isotricha* spp. (37.5% archaea (+)) *P. multivesticulatum* (archaea (−)) *Epidinium* spp. (16.3% archaea (+)) *Eu. maggii* (8% archaea (+)) *Entodinium* spp. (42.8% archaea (+))	5 different types of fauna	- Sheep - Hay (*ad libitum*) + pelleted concentrate/d - Before feeding	[24]
FISH probing	FISH was applied to detect and quantify the associated methanogens in *Entodinium* spp..	Methanogens including *Mbb. thaueri*, *Mbb.millerae* and *Mbb. smithii*, and members of Mm. and *Methanospaera* spp. were generally the predominant colonizers of protozoa. *Entodinium* spp. were colonized by similar methanogenic populations regardless of the forage fed.	*Entodinium* spp.	Cattle - Alfalfa hay or triticale straw - After feeding (1–2 h)	[36]

^a^Mbb = Methanobrevibacter

^b^Mm = Methanomicrobium

^c^RCC = rumen cluster C

**Fig. 2:**
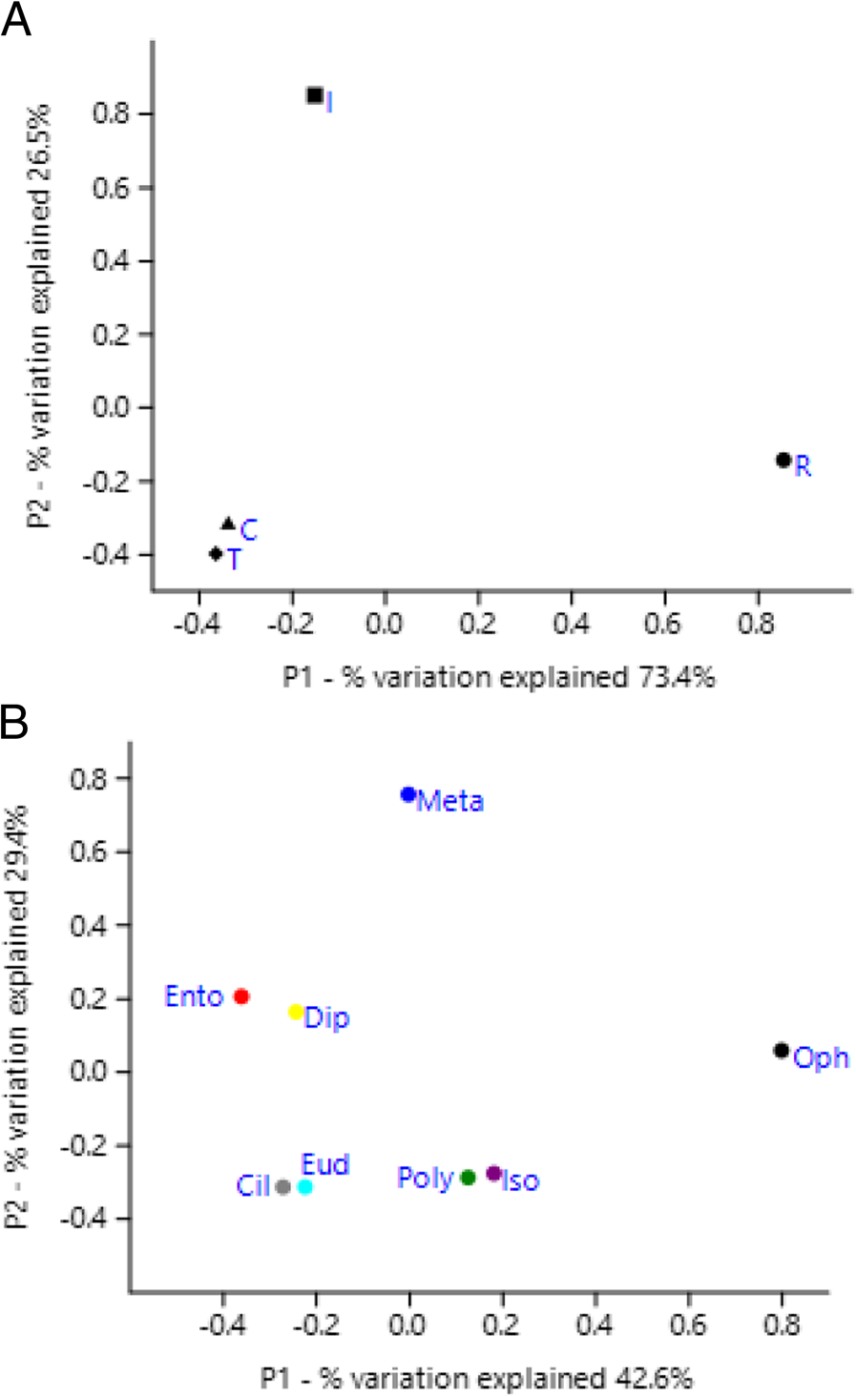
Principal coordinates analysis (PCoA) of 16S rRNA PAM gene sequences obtained from two different studies (**a**) and different rumen ciliate species (**b**). C = Chagan et al. [28]; T = Tokura et al. [29]; I = Irbis & Ushida [30]; R = Regensbogenova et al. [31]. Cil = Ciliate fraction; Dip = *Diplodinium*; Ento = *Entodinium*; Eud = *Eudiplodinium*; Iso = *Isotricha*; Meta = *Metadinium*; Poly = *Polyplastron*; Oph = *Ophryoscolex*. Analyses were conducted using 47 sequences recovered from washing single or several ciliate cells based on the Jukes-Cantor model [177] using MEGA6 [178]

One T-RFLP analysis showed that ruminal protozoa have similar density of methanogens as rumen fluid [17]. Because T-RFLP is not a quantitative method, qPCR will be required to improve the estimate. Early studies based on comparisons in methanogen diversity between faunated and defaunated rumen suggest the impact of rumen protozoa on methanogen diversity and population dynamics [32, 34, 35], but that differences cannot be solely attributed to the PAM. From a sequence-based analysis of washed protozoal cells, different archaeal phylotypes were found to be associated with specific species or genera of protozoa, but discrepancies arose from different studies (Fig. 2). A recent study using FISH, however, showed similar composition and relative abundance of methanogens colonizing ciliates related to *Entodinium simplex*, *E. caudatum*, and *E. furca* [36]. Rumen ciliates were thought to select their prey (primarily bacteria and methanogens) [26, 37], but a recent in vitro study using single species of ruminal protozoa (*E. caudatum*, *Diplodinium dentatum*, and *Metadinium medium*) and ruminal bacteria (*Ruminococcus albus* and *Streptococcus bovis*) showed no selective predation [38]. Conflicting results among studies reflect the difficulties in analysis of true PAM. Selective association between rumen ciliates and methanogens is a fascinating theory. Conceivably, some of the engulfed bacteria and methanogens can escape digestion from the food vacuoles and establish themselves as endosymbionts. It is not known, however, what attributes allow certain methanogens to establish themselves as endosymbionts. Single cell genomics will provide opportunities to further identify PAM and the processes by which PAM is established. Axenic cultures of rumen ciliates will ultimately provide the unequivocal evidence of PAM and help elucidate the mechanisms underpinning their endosymbiosis. However, no axenic culture of rumen ciliates has been achieved despite repeated efforts, suggesting the necessity of a symbiotic relationship with prokaryotes for the long-term survival of ruminal ciliates.

## Interaction of methanogens with other rumen microbes

Some rumen methanogens can also become associated with fungi, but little is known about fungi-associated methanogens (FAM). An early study suggests that rumen fungi do not have endosymbiotic methanogens although they may have ectosymbiotic methanogens [39]. In a recent study, species of *Methanobrevibacter* were detected by PCR in cultures of *Piromyces*, *Anaeromyces*, and *Neocallimastix* [40]. Methanogens were also detected in some rumen fungal cultures, but it was not reported if the methanogens and fungi had any physical association, either ectosymbiotic or endosymbiotic [41, 42]. All rumen fungi contain hydrogenosomes [19, 43], but definitive evidence is needed to determine if rumen fungi carry true endosymbiotic methanogens.

No symbiotic association between rumen bacteria and methanogens is expected, but the integration of methanogens into bacterial biofilms on feed particles in itself represents a form of interaction, and most fermentative ruminal bacteria produce CO_2_ and H_2_, which are the substrates for methanogens [44]. Thus, rumen bacteria and methanogens interact mutualistically through interspecies H_2_ transfer. Such interspecies H_2_ transfer was demonstrated in co-cultures of methanogens with *Ruminococcus albus* [45], *R. flavefaciens* [46], and *Selenomonas ruminantium* [47]. The interaction between rumen bacteria and methanogens affects energy conservation, VFA profiles, and CH_4_ production by the rumen microbiome. More studies are required to investigate microbial interaction at microbiome level. Metagenomic and metatranscriptomic analysis can help determine co-occurrence patterns, which can shine new light on bacteria-methanogen interaction at microbiome level.

## Effects of anti-methanogenic compounds on rumen methanogens

Numerous CH_4_ mitigation technologies have been explored, including interventions of animal management, dietary composition, rumen fermentation, and methanogens [10, 48, 49]. Among these mitigation options, inhibiting the growth or the metabolic activity of methanogens is the most effective approach. Another strategy is to modulate rumen microbiome so that fermentation is shifted toward decreased H_2_ production and increased production of reduced VFA (e.g., propionate). Even though many studies have been reported in the literature, substantial discrepancies exist among different studies concerning the magnitude of efficacy and adverse impact on feed digestion and fermentation. Here we review the anti-methanogenic compounds evaluated with a focus on their impact rumen methanogens.

### Coenzyme M analogs

Methyl-CoM reductase (Mcr) mediates the final step of all the methanogenesis pathways and CoM (2-mercaptoethanesulfonic acid) is an essential cofactor serving as the methyl group carrier. Mcr reduces methyl-CoM to CH_4_. CoM is found in all known methanogens but not in other archaea or bacteria [50]. Several halogenated sulfonated compounds, including 2-bromoethanesulfonate (BES), 2-chloroethanesulfonate (CES), and 3-bromopropanesulfonate (BPS), are structural analogs of CoM, and they can competitively and specifically inhibit Mcr activity, lowering CH_4_ production at relatively low concentrations [51]. Different species of methanogens vary in sensitivity to these inhibitors. Of three species tested on BES, *Mbb. ruminantium* was the most sensitive, while *Methanosarcina mazei* was the least sensitive, with *Methanomicrobium mobile* being intermediate [52]. All three species appeared to be resistant to BPS up to 250 μmol/L in pure cultures [52]. The different sensitivity to these CoM analogs has been attributed to varying ability to uptake these inhibitors into the cells [53, 54]. Methanogens able to synthesize their own CoM are less dependent on external CoM and are thus less sensitive. *Mbb. ruminantium* is the only ruminal methanogen that requires CoM synthesized by other methanogens [55]. Some methanogens can become adapted to BES [52], suggesting that administration of BES could increase growth and persistence of BES-resistant methanogens [56], which is consistent with the limited efficacy of BES in lowering CH_4_ production by rumen microbiome [57]. Thus, halogenated sulfonated compounds probably have limited application to mitigate CH_4_ production at the farm level.

### Halogenated aliphatic C_1_-C_2_ hydrocarbon

Halogenated aliphatic compounds with 1 or 2 carbons, such as chloroform, bromochloromethane (BCM), bromoform, bromodichloromethane, dibromochloromethane, carbon tetrachloride, trichloroacetamide, and trichloroethyladipate, can lower ruminal CH_4_ production [48]. These halogenated compounds block the function of corrinoid enzymes and inhibit cobamide-dependent methyl group transfer in methanogenesis [58]. These halogenated compounds also competitively inhibit CH_4_ production by serving as terminal electron (e^−^) acceptors [59]. Drenching chloroform to cattle inhibited methanogenesis substantially for up to 32 days without affecting feed digestion or basic rumen function, but thereafter the inhibition faded away [60]. The population of RCC increased with time and *Mbb. ruminantium*-related methanogens tended to become more prevalent later in the recovery phase, but methanogen diversity decreased [60]. The addition of BCM depressed CH_4_ production both in vitro [61] and in vivo [62, 63]. In steers fed grain-based diets, BCM decreased CH_4_ production by 50 to 60% with no signs of toxicity or residues in meat [62]. Goel et al. [61] reported that the abundance of total bacteria and protozoa was not changed, but methanogenesis and growth of methanogens were drastically inhibited by BCM in both batch cultures and continuous fermenters. In contrast, BCM did not reduce the abundance of bacteria, protozoa, or methanogens in goats over 57 days although CH_4_ production decreased by 33% [63]. However, the archaeal community structure was altered [63] probably due to adaptation to BCM and/or selection of BCM-resistant methanogens. Therefore, halogenated aliphatic hydrocarbons have a limited utility to mitigate CH_4_ emission at farms. For example, they can deplete ozone and thus they are banned from commercial use in many countries. Chloroform is also a recognized carcinogen. Also, chloroform inhibits homoacetogenic bacteria and acetate-consuming sulfate-reducing bacteria [64]. Although these two groups of bacteria do not have a major role under normal dietary conditions, homoacetogenic bacteria may become important when methanogens are inhibited. Nevertheless, halogenated aliphatic hydrocarbons are not likely to be used on farms to mitigate CH_4_ mitigation because regulatory hurdles will be encountered when these compounds are registered for commercial use.

Some marine plants such as red seaweed, and algae, lichen, and fungi can contain high concentrations of organobromine compounds such as bromomethane and bromoform [65]. A recent in vitro study showed that red seaweed *Asparagopsis taxiformis* lowered CH_4_ production by 99% at a dose of 2% of organic matter substrate [66]. No adverse effect on feed digestion or fermentation was noted at ≤5% (of dry matter) inclusion. Thus, red seaweed, and probably other organobromine-rich plants, may offer a potentially practical natural approach to mitigate CH_4_ emission. In vivo studies are required to determine optimum doses and to evaluate the effect on rumen microbiome, feed fermentation, as well as possible toxic effects. Moreover, large-scale production and transportation of these products to mitigate enteric methane emissions globally will also present a challenge.

### Nitrooxy compounds

3-Nitrooxypropanol (3NOP) and ethyl-3NOP, two new synthetic compounds, have been shown to have specific anti-methanogenic properties. 3NOP appears to inactive Mcr by competitively binding to the Mcr active site and then oxidizing the Ni^1+^ that is required for Mcr activity [67]. The efficacy of 3NOP in lowering CH_4_ production varies considerably. Feeding of 3NOP at a dose rate of 2.5 g/day/cow mixed in diets decreased CH_4_ emission by 60% per kg of DM intake [68]. In a study using beef cattle, 3NOP fed at 2.0 g/day/cow decreased CH_4_ yield by 59%, and the inhibition persisted for up to 112 days without much effect on feed intake, nutrient digestibility or total VFA concentrations [69]. In one recent study [9], 3NOP fed at 40–80 mg/kg feed DM in dairy cows decreased CH_4_ production by about 30% persistently for up to 84 days. Similarly, 3NOP fed at 2.5 g/day/cow decreased CH_4_ yield by 37% in dairy cows [70]. In sheep, 3NOP at 0.5 g/day also decreased CH_4_ production by 29% without adverse effect on digestion or rumen fermentation [71]. However, when 3NOP was directly added to the rumen through rumen cannula at a daily dose of 0.50 or 2.5 g per cow (equivalent to﻿ 25 to 125 mg/kg feed dry matter), the degree of CH_4_ suppression declined to 7–10% [72]. The later study suggests that 3NOP needs to be fed together with the diet to achieve efficacy. It seems that 3NOP could be used to lower CH_4_ emission from cows and sheep without adverse effects on nutrient utilization or animal performance. Only one study examined the effect of 3NOP on rumen methanogens, and it showed that 3NOP decreased methanogen abundance while increasing that of protozoa [69]. Future studies are warranted to investigate how 3NOP affects methanogens and other rumen microbes.

### Pterin compounds

Pterin is a group of structural analogs of deazaflavin (F_420_), which is a coenzyme involved in two steps of the hydrogenotrophic methanogenesis pathway [73]. Therefore, pterin compounds can competitively inhibit CH_4_ production. In one study, CH_4_ production by *Mbb. ruminantium*, *Ms. mazei*, and *Mm. mobile* was significantly decreased by lumazin (2,4-pteridinedione) in a dose-dependent manner from 0.06 to 0.24 mmol/L [52]. As expected, pterin is much less efficacious in mixed rumen cultures than in pure methanogen cultures [52]. It was suggested that lumazine could be degraded or transformed by some microbes in mixed cultures or adsorbed to solid particles where it becomes unavailable to methanogens. Some N-substituted derivatives of *p*-aminobenzoic acid, which are inhibitors of methanopterin synthesis in methanogens, decreased methanogenesis in ruminal cultures without inhibiting VFA production [74]. *Mbb. ruminantium* appeared to be able to adapt to low concentrations of this pterin compound over time, while *Ms. mazei* and *Mm. mobile* could not. Apparently, methanogens vary in susceptibility to pterin. It remains to be shown if pterin affects the diversity of methanogens and other rumen microbes.

### Hydroxymethylglutaryl-CoA (HMG-S-CoA) reductase inhibitors

All archaea contain long-chain isoprenoid alcohols as the major component of their cell membrane [75]. Isoprenoid alcohols are unique to archaea. They are synthesized from mevalonate that is formed by reduction of 3-hydroxy-3-methylglutaryl coenzyme A (HMG-S-CoA) catalyzed by HMG-S-CoA reductase. This enzyme is also used for the synthesis of the same precursor ultimately used in cholesterol synthesis in humans. As an inhibitor of HMG-S-CoA reductase, statins can inhibit the growth of methanogens by inhibiting the synthesis of mevalonate [76]. Lovastatin and mevastatin may also act as a potential inhibitor of F_420_-dependent NADP oxidoreductase as shown in the model structure of that enzyme [77]. In the earliest reported study, mevastatin at 5.6 μmol/L inhibited the growth of all three strains of rumen *Methanobrevibacter*, but not rumen bacteria in vitro [78]. In studies using a rumen simulation technique (Rusitec), lovastatin (150 mg/L) reduced CH_4_ production by approximately 42% without altering bacterial counts or nutrient fermentation [79]. Statins (e.g., lovastatin and mevastatin) are expensive prescription drugs to lower cholesterol in humans [80]. The high cost makes statins cost-prohibitive as anti-methanogenic inhibitors.

The high cost of pure statins promoted a search for natural sources of statins as agents to mitigate CH_4_ production. Lovastatin is a secondary metabolite of idiophase of several fungal species (e.g., *Penicillium* spp., *Aspergillus terreus*, *Monascus purpureus*, and *Pleurotus ostreatus*), and it can reach a concentration up to 2.8% of the dry weight of *P. ostreatus* (oyster mushrooms) [81] and 261 mg/kg fermented rice straw [82]. Lovastatin extracted from fermented rice straw significantly reduced total CH_4_ production by rumen methanogens by nearly 28% after 48 h in vitro incubation [82]. Extract from *A. terreus*-fermented rice straw containing lovastatin (97 mg/g dry mass) also significantly reduced CH_4_ production and abundance of methanogens, especially *Methanobacteriales*, and aerobic fungi, but increased several fiber-degrading bacteria [82]. Lovastatin also altered the morphology of *M. smithii* significantly, resulting in abnormal membrane formation and asymmetric cell divisions and increased HMG-S-CoA reductase gene expression [83]. Fermented rice straw extract also modulated expression of several genes associated with methanogenesis, increasing expression of *mtr*, *mta*, and *mcr* while decreasing expression of *hmd* and *fno* [83]. Supernatant fractions containing statins produced by *Mortierella wolfii* also appeared promising to inhibit methanogenesis without reducing overall fermentation [84]. In another study using sheep, fermented rice straw containing metabolites (possibly pravastatin and mevastatin) produced by *Monascus* spp. decreased CH_4_ emission (by 30%), the abundance of methanogens, and ruminal acetate: propionate ratio compared with the unfermented rice straw [85]. If these fungi could be grown on low-quality forages such as straws, they can be used to decrease CH_4_ production in ruminants. However, many fungi produce mycotoxins, which must be avoided for the practical feeding of animals [86].

Diallyl disulfide, the main ingredient of garlic oil, is known to inhibit HMG-S-CoA reductase [87]. Garlic oil (300 mg/L) was more effective than lovastatin as an inhibitor of CH_4_ production (by up to 91% reduction); however, garlic oil also inhibited bacterial growth, which likely reduces the availability of methanogenesis substrates [79]. Garlic oil lowered CH_4_ production in vitro and growth of methanogens, altered community structure of methanogens after 24 h incubation [7, 8]. Moreover, interestingly, the anti-methanogenic efficacy increased over time up to 18 days of incubation [88]. Few studies have tested garlic oil in vivo. In one study using sheep, neither diallyl disulfide nor lovastatin decreased CH_4_ emission per animal, but both treatments modestly reduced CH_4_ produced per g of dietary fiber consumed [89]. Feeding of garlic or its metabolites may influence the flavor of meat and milk from ruminants [90].

### Anti-methanogen vaccines

Upon vaccination, anti-methanogen antibodies were found in the serum of vaccinated sheep [91]. The first two anti-methanogen vaccines were prepared from whole cells of three and seven selected methanogens in Australia, and these vaccines resulted in no or minimal (only 8% compared to control) decrease in CH_4_ emission [92]. The inefficacy was attributed to the small numbers of methanogen species that the vaccines could target. However, methanogen abundance or CH_4_ production was not decreased by vaccination using a vaccine that was based on a mixture of five methanogen species representing >52% of the rumen methanogen populations, though the composition of methanogens was altered [93]. It was suggested that anti-methanogen vaccines should be developed based on cell surface proteins that are conserved among rumen methanogens to achieve effective results [94]. It should be noted that most antibodies circulate in the blood of a host, and only a tiny amount can enter the rumen through saliva. The amount of antibodies entering the rumen is probably too small to have any effect. Also, antibodies entering the rumen can be rapidly degraded by proteolytic bacteria therein. It stands to reason that vaccination may not be a feasible approach to mitigate CH_4_ emission from livestock.

### Fats and fatty acids

Feeding fat to ruminants lowers CH_4_ emissions [95, 96]. Based on a meta-analysis, fat supplementation reduced CH_4_ by 3.77% in cattle and 4.30% in sheep per 1% dietary fats [97, 98]. Fat decreases CH_4_ production (expressed as g/kg digestible DM) more from sheep than from cattle, which was attributed to the comparatively lower depression of DM digestion together with numerically larger depression of CH_4_ production (g/kg DM) by fat in sheep [98]. Among fatty acids, C12:0, C18:3 and other polyunsaturated fatty acids (PUFA) are more potent than saturated fatty acids [97, 99]. The CH_4_-suppressing efficacy of fats generally persists [97], with persistent suppression being noted for 72 days and longer [100, 101] in cattle.

Fats supplemented up to 6% of the diet (DM) can also improve milk production while appreciably decreasing CH_4_ emissions (15%) in cattle, but higher concentrations decreased production efficiency due to a reduction of feed digestion and fermentation [97]. Medium-chain fatty acids (MCFA) and PUFA can lower abundance and metabolic activities of rumen methanogens and change their species composition [95, 99, 102]. PUFA can also directly inhibit protozoa and serve as hydrogen sink through biohydrogenation [103]. Both MCFA and PUFA appear to damage the cell membrane, thereby abolishing the selective permeability of cell membrane, which is required for survival and growth of methanogens and other microbes [104]. The inhibitory effect of fat on methanogenesis is more pronounced in cattle fed concentrate-based diets than in cattle fed forage-based diets [97]. Because C12: and C14:0 is more inhibitory to *M. ruminantium* at pH 5 than at pH 7 [105], the concentrate level-dependent anti-methanogenic efficacy of MCFA and PUFA is probably attributed to the lower pH associated with high-concentrate diets.

### Plant secondary metabolites

Plants secondary metabolites (PSM), such as saponins, tannins, flavonoids, organosulphur compounds, and essential oils, have anti-microbial activities against several types of microorganisms [106]. Numerous PSM extracts have been recognized as potential inhibitors of rumen methanogens and CH_4_ production [107, 108]). Some forage plants rich in tannins and saponins have also shown promise at mitigating CH_4_ emission from ruminants [109, 110]. However, the efficacy of PSM in suppressing CH_4_ production varies considerably depending on the type, sources, molecular weight, doses, as well as diet types.

A) *Tannins*

Tannins decrease CH_4_ production by directly inhibiting methanogens and indirectly decreasing H_2_ production as a result of decreased fiber digestion and protozoal population in the rumen [48]. The inhibitory activity of tannins extracted from *Lotus pedunculatus* was demonstrated on pure cultures of methanogens [111]. Puchala et al. [109] also showed inhibition of methanogen populations by tannins in the rumen of goats fed diets containing tannins. Studies on structure-activity relationships have shown that types and molecular weights of tannins are important in determining their potency in lowering CH_4_ production and abundance and diversity of rumen methanogens, with high molecular weight condensed tannins (CT) being more potent [112, 113]. Such structure-activity relationships have been demonstrated using members of *Methanobacteriales* including *Methanobrevibacter* [114]. However, members of *Methanomicrobiales* exhibit no differential response to CT with different molecular weights, and unclassified *Thermoplasmata*-associated methanogens were even stimulated with increasing molecular weights of CT [114]. One of the CT fractions also increased the relative abundance of *Methanomicrobium* spp. The differential responses of methanogens to different CT and variation in the CT used among studies may explain the inconsistent effects reported despite using similar doses of tannins.

B) *Flavonoids*

Flavonoids have not been extensively evaluated with respect to rumen methanogenesis [107]. Oskoueian et al. [115] reported that inclusion of flavone, myricetin, naringin, rutin, quercetin, or kaempferol decreased in-vitro CH_4_ production by 5 to 9 mL/g DM. Their potency ranked as follows: myricetin ≥ kaempferol ≥ flavone > quercetin ≥ naringin > rutin ≥ catechin. Catechin decreased CH_4_ production both in vitro [116] and in vivo [117]. All the flavonoids, when fed at 0.2 g/kg DM, noticeably decreased relative abundances of hydrogenotrophic methanogens, and citrus (*Citrus aurantium*) extract rich in mixed flavonoids and its pure flavonoid components, neohesperidin and naringin, appeared to result in the greatest inhibition [118]. *Methanosarcina* spp. were also inhibited by poncirin, neohesperidin, naringin and their mixture. Flavonoids directly inhibit methanogens [115, 118] and also likely acts as H_2_ sinks via cleavage of ring structures (e.g., catechin) and reductive dihydroxylation [116].

C) *Saponins*

The effects of saponins on rumen fermentation, rumen microbial populations, and ruminant productivity have been examined extensively and reviewed previously [107, 108, 119]. *Quillaja* saponin at 1.2 g/L, but not at 0.6 g/L [120], lowered CH_4_ production in vitro and the abundance of methanogens (by 0.2–0.3 log) and altered their composition. Ivy fruit saponin decreased CH_4_ production by 40%, modified the structure of the methanogen community, and decreased its diversity [121]. Saponins from *Saponaria officinalis* decreased CH_4_ and abundance of both methanogens and protozoa in vitro [122]. However, in other in vitro studies, *Quillaja* saponins at 0.6 g/L did not lower CH_4_ production or methanogen abundance [88, 123], and *Yucca* and *Quillaja* saponins at 0.6 to 1.2 g/L even increased archaeal abundance (by 0.3–0.4 log), despite a decrease in protozoal abundance by *Quillaja* saponin [124]. Tea saponins (30 g/day) also did not lower CH_4_ emission from steers or abundance of total methanogens but increased the abundance of RCC methanogens and protozoa [125]. Thus, the effects of saponins on methanogenesis and methanogen abundance are highly variable among studies.

Saponins probably have little direct effect on methanogens but are known to inhibit rumen protozoa, lowering H_2_ production and decreasing the abundance of PAM [126]. It has been estimated that PAM produce 9–25% [127] or more (37%) of total CH_4_ production [21] in sheep. The difference in PAM and their proportion of total methanogens, diet composition, and dose and chemical nature of saponins can be attributable to the discrepancies among studies.

D) *Essential oils*

The effects, mostly beneficial, of essential oils (EO) on rumen fermentation, microbial populations, and ruminant productivity have frequently been reviewed [108, 128–130]. Several EO compounds, either in pure form or in mixtures, are anti-methanogenic [123, 131–133]. The effects of EO on CH_4_ production and methanogens are variable depending on dose, types, and diet. Patra and Yu [7] compared five EO (clove, eucalyptus, peppermint, origanum, and garlic oil) that have different chemical structures in vitro at three different doses (0.25, 0.50 and 1.0 g/L) for their effect on CH_4_ production and archaeal abundance and diversity. Overall, all these EO suppressed CH_4_ production and abundance of archaea and protozoa in a dose-dependent manner, but they differed in potency. Thyme oil or cinnamon oil fed to Holstein steers at 0.5 g/day decreased the relative abundance of total protozoa and methanogens [134]. However, feeding beef cattle a blend of EO (CRINA®) did not affect CH_4_ production, methanogen abundance or its diversity [135]. Overall, methanogens may be directly inhibited or indirectly inhibited by EOs via inhibition of protozoa and H_2−_producing bacteria in the rumen [130, 131].

### Alternative hydrogen sinks

Compounds with a redox potential higher than CO_2_ can thermodynamically outcompete CO_2_ for reducing equivalents produced during rumen fermentation. These compounds, thus, can be used as alternative e^−^ acceptors to redirect e^−^ flux away from methanogenesis. The commonly evaluated alternative e^−^ acceptors are discussed below.

A) *Nitrate and sulfate*

Nitrate (NO_3_^1−^) decreased CH_4_ production both in vitro [120, 136, 137] and in vivo [138–141]. Mechanistically, nitrate decreases CH_4_ production by outcompeting CO_2_ as an e^−^ acceptor, and its reduction intermediates, nitrite (NO_2_^1−^) and nitrous oxide (N_2_O), also directly inhibit methanogens as well as some H_2_ producers [8, 120, 142, 143]. Sulfate also lowers CH_4_ production, but much less effectively than nitrate. Archaeal abundance declined in goats receiving nitrate [144]. While nitrate is not toxic to methanogens, it is toxic to protozoa, fungi and to a lesser extent to select bacterial species, suggesting a more general toxicity of nitrate [143]. Nitrate can replace a portion of the dietary nitrogen as it is reduced to ammonia. However, dietary nitrate supplementation may increase the risk of nitrite poisoning (methemoglobinemia), especially when forage contains a high level of nitrate [136]. High concentrations of sulfate in diets (i.e., 0.3 to 0.4% sulfur as sulfate) can reduce feed intake, animal performance, and increase the risk of sulfur-associated polioencephalomalacia [145].

B) *Nitrocompounds*

A few organic nitrocompounds have been evaluated for their efficacy to decrease methanogens and CH_4_ production as recently reviewed by Latham et al. [146]. These compounds can serve as e^−^ acceptors by some bacteria competing with methanogens for reducing equivalents. This is demonstrated by nitroethane that can be used as a terminal e^−^ acceptor by *Dentitrobacterium detoxificans*, thereby indirectly decreasing CH_4_ production [146, 147]. Nitrocompounds may also inhibit methanogenesis by directly inhibiting the activity of formate dehydrogenase/formate hydrogen lyase and hydrogenase, all of which are involved in the early step(s) of the hydrogenotrophic methanogenesis pathway [148], or inhibiting e^−^ transfer between ferredoxin and hydrogenase [146]. However, these premises have not been confirmed biochemically.

Nitrocompounds generally are quite effective in lowering CH_4_ production, with 3-nitro-propionate, 2-nitropropanol, 2-nitroethanol and nitroethane being able to decrease CH_4_ production by 57 to 98% in vitro [148]. Using sheep, Anderson et al. [147] showed that nitroethane decreased CH_4_ production by up to 45% and 69%, respectively, when orally administrated at 24 and 72 mg/kg body weight daily for 5 days. Although less effective than nitroethane, 2-nitropropanol also significantly lowered CH_4_ production (by 37%) in steers. However, the effect of both nitroethane and 2-nitropropanol diminished at day 5 of administration, presumably due to microbial adaptation. In another study, daily oral administration of nitroethane up to 160 mg/kg BW failed to lower CH_4_ emissions in steers, and microbial transformation and adaptation were thought to be responsible for the lack of persistent efficacy [149]. Nitroethane or 2-nitropropanol generally have no effect on rumen fermentation, but due to rapid adaptation by rumen microbes, they are probably of little practical utility in methane mitigation.

C) *Propionate and butyrate enhancers*

Malate, acrylate, oxaloacetate, and fumarate are intermediates of carbohydrate fermentation. They can be converted to propionate or used in anabolism for the synthesis of amino acids or other molecules. They can accept reducing equivalents and thus stoichiometrically lower H_2_ available for CH_4_ production. When added at a concentration of 3.5 g/L, fumarate decreased CH_4_ production by 38% in continuous fermenters with forages as a substrate [150]. Types of forages and their combinations appeared to affect the anti-methanogenic efficacy of fumarate, ranging from 6 to 27% inhibition at 10 mmol/L [151]. Acrylate also depresses CH_4_ production in the rumen, but to a lesser extent than an equimolar level of fumarate. Malate was found to decrease CH_4_ production by beef cattle in a dose-dependent manner, with a 16% decrease being noted when fed at 7.5% of DM intake, which corresponds to a 9% reduction per unit of DM intake [152]. Different studies reported different anti-methanogenic potencies of this type of e^−^acceptors. Fumarate fed to goats at 10 g/day/goat was found to decrease the abundance of methanogens and CH_4_ production only by 11.9% while increasing concentrations of total VFA, acetate and propionate [153]. However, CH_4_ emissions were not lowered by tartrate, malate, fumarate, or citrate at up to 15 mmol/L in vitro [154], or by oxaloacetate at up to 18 mmol/L in vitro [155]. Fumarate fed at up to 29 g/kg DM did not decrease CH_4_ emission from beef cattle [156]. Some of the intermediates of pyruvate conversion to butyrate can act as e^−^ acceptors, which could also decrease CH_4_ production. Ungerfeld et al. [155] evaluated acetoacetate, β-hydroxybutyrate, and crotonic acid at up to 18 mmol/L in vitro. β-Hydroxybutyrate did not lower CH_4_ production, while the other two intermediates only decreased CH_4_ production by ≤18%. The inconsistent efficacies reported in the literature can be attributed to many factors, including variation in diet used and type and dose tested. Besides, these intermediates can be converted to acetate, rather than propionate or butyrate, thereby stoichiometrically increasing CH_4_ production [157]. Nonetheless, it is probably cost-prohibitive to use these organic acids to lower CH_4_ emissions on farms.

D) *Unsaturated organic acids*

Unsaturated fatty acids can act as hydrogen sinks during their biohydrogenation and thereby lower CH_4_ production. Propynoic acid (an unsaturated analog of propionic acid), 3-butenoic acid and 2-butynoic acid (both unsaturated analogs of butyric acid), and ethyl 2-butynoate each at 6 to 18 mmol/L have been evaluated as alternative e^−^ sinks to lower methanogenesis in vitro [155]. Only propynoic acid and ethyl 2-butynoate markedly lowered CH_4_ production, by 65 to 76% and 24 to 79%, respectively [155]. In another study, propynoic acid lowered CH_4_ production by 67% and 78% at 6 and 12 mmol/L, respectively and decreased methanogen abundance [120]. Propynoic acid and ethyl 2-butynoate are directly toxic to methanogens, and species of methanogens vary in their sensitivity to these two inhibitors, with *Mbb. Ruminantium* being most sensitive, *Ms. mazei* least sensitive, and *Mm. mobile* intermediate [52]. The S-layer in *Ms. Mazei* and *Mm. mobile* (absent in *Mbb. ruminantium*) may confer some resistance to propynoic acid and ethyl 2-butynoate. Selective resistance to these compounds among different species can favor the proliferation of resistant species over time, diminishing any initial decreases in enteric CH_4_ production, which makes it ineffective to use these inhibitors in vivo.

### Inhibitors to hydrogen-producing bacteria

A) *Ionophores*

Ionophores, such as monensin and lasalocid, are commonly used to improve rumen microbial metabolism. Being highly lipophilic ion carriers, they pass through the cell wall of Gram-positive bacteria and penetrate into the cell membrane. Therein, they serve as H^+^/Na^+^ and H^+^/K^+^ antiporters, dissipating ion gradients that are needed for ATP synthesis, nutrient transport, and other essential cellular activities and ultimately resulting in delayed cell division and even cell death [158]. Ionophores preferentially inhibit Gram-positive bacteria, including members of class *Clostridia*, including *Ruminococcus* species that produce acetate and H_2_ [159]. Ionophores can also inhibit some Gram-negative rumen bacteria [160, 161], including bacteria that produce formate and H_2_ [159]. Therefore, ionophores may lower CH_4_ emission by decreasing H_2_ production. For examples, monensin fed at 24–35 mg/kg diet lowered CH_4_ production by up to 10% (g/kg DM intake) [162–165], though no CH_4_ suppression was observed at 10–15 ppm. In a recent in vivo study, however, monensin at 60 mg/day/cow did not lower CH_4_ production by tropical cattle, though it decreased CH_4_ production by about 30% when fed at 250 mg/day/cow [135]. As repeatedly noted, at such high supplementation level, DM intake was lowered, which explains most of the observed decrease in CH_4_ emission. Ionophores are not known to directly inhibit methanogens, but they can change the population dynamics of methanogen species. For example, monensin decreased the population of *Methanomicrobium* spp. while increasing that of *Methanobrevibacter* spp. [135]. Total methanogens were also decreased in cattle fed monensin [134]. These can be explained by reduced availability of H_2_and differences in affinity for H_2_ and growth kinetics among methanogen species.

B) *Bacteriocins*

Bacteriocins are proteins or peptides produced by bacteria and inhibit select microbial species in the rumen and other habitats. There are only a few studies investigating the effect of bacteriocins on CH_4_ emission. Bovicin HC5, a bacteriocin produced by *Streptococcus* spp. from the rumen, was reported to suppress CH_4_ by 50% in vitro [166]. Nisin, a bacteriocin produced by *Lactobacillus lactis* subsp. *lactis*, has also been shown to decrease CH_4_ production in vitro by up to 40% depending upon its concentration [167]. Similar to monensin, bacteriocins probably modulate rumen fermentation leading towards increased propionate, thereby decreasing CH_4_ production. Bacteriocins may hold some potential in mitigating enteric CH_4_ emission, but further research is needed to confirm their efficacy in vivo and to determine their cost.

### Use of combination of anti-methanogenic inhibitors

Most of the aforementioned anti-methanogenic inhibitors have repeatedly been evaluated, primarily individually, both in vitro and in vivo, to decrease enteric CH_4_ production. With a few exception (e.g., nitrate and 3NOP), most of them often decrease feed intake, feed digestion, and rumen fermentation when added at high enough doses to achieve effective CH_4_ inhibition [120]. Some of these inhibitors (e.g., halogenated aliphatic hydrocarbons) are also toxic to animals [168]. Adverse effects or toxicity can be avoided by using combinations of inhibitors with complementary modes of actions at low doses to inhibit not only methanogens but also other rumen microbes (e.g., protozoa and H_2_-producing bacteria) that contribute to CH_4_ production in an additive or synergistic manner [120]. Binary combinations of nitrate and *Quillaja* saponin inhibited CH_4_ production additively in vitro (by 32% at 5 mmol nitrate/L and 0.6 g/L saponins, and by 58% at 10 mmol nitrate/L and 1.2 g/L saponins) and decreased the abundances of methanogens without affecting feed digestion or fermentation [120]. This binary combination probably additively lowered CH_4_ production by channeling H_2_ away from methanogenesis to nitrate reduction, directly inhibiting methanogens by nitrite (the intermediate of nitrate reduction), and inhibiting protozoa and their PAM [120]. Combinations of garlic oil and nitrate, garlic oil and nitrate and saponin, and saponin and nitrate and sulfate all considerably decreased CH_4_ production and abundance of methanogens and altered the species composition of methanogens in vitro without other adverse effects [8, 88, 169]. Monensin in combination with ethanol extract of hops (*Humuluslupulus*, containing β- and α-acids) or *Yucca* saponin decreased CH_4_ in an additive manner in vitro, but unfortunately, microbial protein synthesis was also decreased [170]. Use of a combination of different anti-methanogenic inhibitors is a relatively new approach, and only a few in vivo studies have been reported. Combinations of nitrate and sulfate additively lowered CH_4_ production in sheep without decreasing feed digestibility or fermentation [8, 120, 142, 143]. These were also observed in dairy cows when fed combinations of nitrate and linseed oil [171]. Because CH_4_ production in the rumen involves methanogens and several groups of other microbes, combinations of inhibitors with complementary modes of actions represent a paradigm shift in achieving effective and practical CH_4_ mitigation from ruminants. Future research can help optimize combinations and doses to achieve sustainable and practical CH_4_ mitigation from ruminants.

## Concluding remarks and future perspectives

Previous research has helped reach a sound understanding and appreciation of the diversity of rumen methanogens in general. However, variations among individual animals are ubiquitous, and the underpinning of such variation is poorly understood. The relationship between animal performance and diversity/population dynamics also remains to be determined and elucidated. Additionally, methanogens associated with protozoa and fungi continue to be elusive, so does their symbiotic relationship with these two groups of eukaryotes and phages. Moreover, it is unknown to what extent phages, both bacterial and archaeal, affect the population dynamics of rumen bacteria and methanogens and thus CH_4_ emission. Because these methanogens and their symbiotic relationship affect fermentation and CH_4_ emission from ruminants, more future research is warranted.

A large number of synthetic and natural compounds have been tested, but most of them exhibited inconsistent efficacy. Variations in rumen microbiome, fermentation kinetics, response and adaptation to anti-methanogenic inhibitors, and diet are probably among the major factors that contribute to the inconsistent efficacy. More importantly from an application perspective, the desired decrease in CH_4_ production often is accompanied by significant reduction in feed intake, digestion, and fermentation. Given that CH_4_ production in the rumen is a multifaceted process involving methanogens as well as many different H_2_-producing microbes, such challenges are expected. Future ecological and physiological research on methanogens and other microbes involved in CH_4_ production can help predict the efficacy of anti-methanogenic compounds. Combinations of anti-methanogenic compounds with complementary modes of actions are a promising approach to achieve effective CH_4_ mitigation without adverse effects on feed intake and rumen fermentation. Mechanistic research on most anti-methanogenic inhibitors lags behind empirical studies. Future mechanistic research will help formulate new combinations and optimize their composition and doses to achieve persistent and effective CH_4_ emission. A cost-benefit assessment of the mitigation options and carbon footprint analysis of the livestock products using an integrated life cycle assessment needs to be done before any CH_4_ mitigation effort can be put into practice. There are also several other challenges in using some of the anti-methanogenic compounds in ruminant production. For some of the anti-methanogenic substances, especially synthetic compounds, it may be difficult to obtain regulatory approval for commercial applications on farms. There are also challenges for the administration of the compounds, especially to ruminants that are under extensive grazing conditions. This is especially important as the cattle grazing pasture make the largest contribution to enteric methane emissions globally.

## Abbreviations

3NOP: 3-nitrooxypropanol
BCM: Bromochloromethane
BES: 2-bromoethanesulfonate
BPS: 3-bromopropanesulfonate
CES: 2-chloroethanesulfoante
CH_4_: Methane
CO_2_: Carbon dioxide
CoA: Coenzyme A
CoM: Coenzyme M
CT: Condensed tannins
e^−^: Electron
EO: Essential oils
F_420_: Deazaflavin
FAM: Fungi-associated methanogens
FISH: Fluorescence in situ hybridization
H_2_: Hydrogen gas
HMG-S-CoA: Hydroxymethylgluaryl-CoA
*Mbb*.: *Methanobrevibacter*
MCFA: Medium-chain fatty acids
Mcr: Methyl-CoM reductase
*Ms*.: *Methanosarcina*
NGS: Next-generation sequencing
PAM: Protozoa-associated methanogens
PSM: Plant secondary metabolites
RCC: Rumen cluster C
RDP: Ribosomal database project
Rusitec: Rumen simulation technique
T-RFLP: Terminal restriction fragment length polymorphism
VFA: Volatile fatty acids
